# Potential for Gut Peptide-Based Therapy in Postprandial Hypotension

**DOI:** 10.3390/nu13082826

**Published:** 2021-08-17

**Authors:** Malcolm J. Borg, Cong Xie, Christopher K. Rayner, Michael Horowitz, Karen L. Jones, Tongzhi Wu

**Affiliations:** 1 Adelaide Medical School and Centre of Research Excellence in Translating Nutritional Science to Good Health, The University of Adelaide, Adelaide 5000, Australia; malcolm.borg@sa.gov.au (M.J.B.); c.xie@adelaide.edu.au (C.X.); chris.rayner@adelaide.edu.au (C.K.R.); michael.horowitz@adelaide.edu.au (M.H.); karen.jones@adelaide.edu.au (K.L.J.); 2 Endocrine and Metabolic Unit, Royal Adelaide Hospital, Adelaide 5000, Australia

**Keywords:** postprandial hypotension, glucagon-like peptide-1, glucose-dependent insulinotropic polypeptide, somatostatin, diabetes mellitus, autonomic failure

## Abstract

Postprandial hypotension (PPH) is an important and under-recognised disorder resulting from inadequate compensatory cardiovascular responses to meal-induced splanchnic blood pooling. Current approaches to management are suboptimal. Recent studies have established that the cardiovascular response to a meal is modulated profoundly by gastrointestinal factors, including the type and caloric content of ingested meals, rate of gastric emptying, and small intestinal transit and absorption of nutrients. The small intestine represents the major site of nutrient-gut interactions and associated neurohormonal responses, including secretion of glucagon-like peptide-1, glucose-dependent insulinotropic peptide and somatostatin, which exert pleotropic actions relevant to the postprandial haemodynamic profile. This review summarises knowledge relating to the role of these gut peptides in the cardiovascular response to a meal and their potential application to the management of PPH.

## 1. Introduction

Postprandial hypotension (PPH) is defined as a fall in systolic blood pressure (SBP) of ≥20 mmHg, or a decrease to ≤90 mmHg if normotensive at baseline, within 2 h of a meal [1]. It is under-recognised, despite occurring frequently in the elderly (prevalence ~20–30%) and individuals with type 2 diabetes mellitus (T2D) (~40%) and chronic neurological disorders, such as Parkinson’s disease (40–100%) [1]. PPH is associated with substantially increased morbidity and mortality, and predisposes to syncope, falls, angina, transient ischaemic attacks and stroke [2]. The pathophysiology underlying PPH remains incompletely understood, but emerging evidence has revealed the fundamental role of gastrointestinal function in the regulation of cardiovascular responses to a meal [1], particularly the secretion and action of gut-derived peptides including glucagon-like peptide-1 (GLP-1) [3], glucose-dependent insulinotropic polypeptide (GIP) [4,5] and somatostatin [6]. Indeed, therapeutic strategies that modulate gastrointestinal hormone secretion or signalling have been shown to influence postprandial blood pressure substantially, although few, if any, studies have assessed whether the available therapies have a sustained effect on postprandial blood pressure or can prevent complications of PPH [1]. This review discusses the relevance of gastrointestinal function to the regulation of postprandial blood pressure, with an emphasis on the role of gut peptides in the pathophysiology and management of PPH.

## 2. Gastrointestinal Regulation of Postprandial Blood Pressure

**Figure 1:**
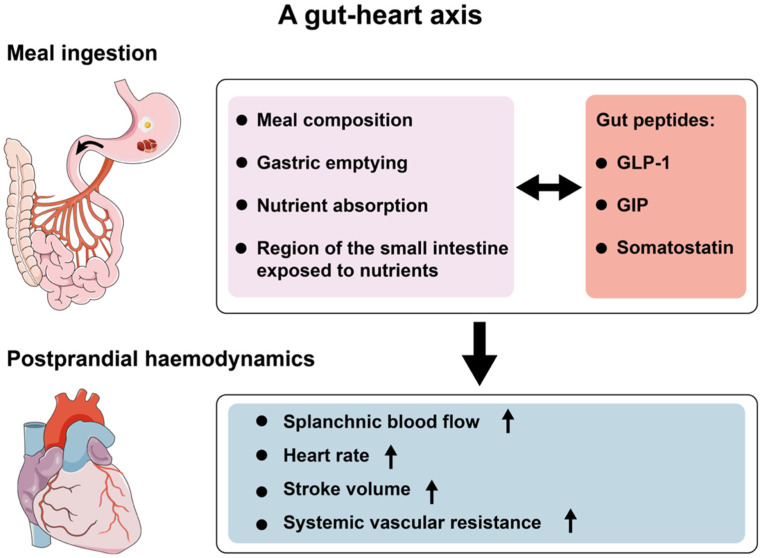
Proposed model by which the gut modulates the postprandial haemodynamic response. The composition of the meal ingested, rate of small intestinal nutrient exposure and subsequent absorption of nutrients, region of small intestine exposed to nutrients and neurohormonal responses all affect postprandial haemodynamics, with the potential to influence the blood pressure response to a meal. Gut peptides, notably GLP-1 [7], GIP [8] and somatostatin [9], may have profound effects on postprandial haemodynamic responses.

The onset of PPH reflects inadequate cardiovascular compensation to meal-induced splanchnic blood pooling, which results from complex interactions between ingested nutrients and the gastrointestinal tract. There is now compelling evidence that gastrointestinal factors, including meal composition, the rate of nutrient delivery to the small intestine (i.e., gastric emptying), nutrient absorption, the specific region of the small intestine exposed to nutrients, and the consequent neurohormonal responses, are integral to the postprandial blood pressure response (Figure 1) [1].

Carbohydrate, fat and protein have all been shown to induce variable haemodynamic responses. For example, in hypertensive older individuals, oral carbohydrate, but not fat or protein, reduced mean blood pressure [10]. In “healthy” older individuals, intraduodenal administration of glucose (3 kcal/min) led to a more rapid decline in SBP when compared with intraduodenal isocaloric fat and protein [11], and the increase in superior mesenteric artery blood flow, a surrogate measure of splanchnic blood pooling, in response to intraduodenal protein was less when compared with glucose and fat [11]. In individuals with T2D, intraduodenal infusion of glucose (2 kcal/min), but not lipid, reduced diastolic blood pressure (DBP) [12]. The variable haemodynamic responses to macronutrients may reflect differences in neurohormonal profiles; for example, fat, relative to isocaloric glucose, is a more potent stimulus for secretion of the two incretin hormones, GLP-1 and GIP [12,13].

The rate of nutrient delivery into the small intestine is tightly controlled by gastric emptying, which exhibits substantial inter-individual, but much less intra-individual, variation [14]. Along with the observation that gastric emptying predicts postprandial glucose excursions [14], changes in postprandial blood pressure have also been found to be related to the rate of gastric emptying. There is now compelling evidence that gastric emptying represents a major determinant of the blood pressure response to a meal, such that the postprandial fall in SBP is greater when gastric emptying is relatively more rapid [15,16], reflecting enhanced small intestinal nutrient interaction and splanchnic blood pooling [17]. However, the relationship between gastric emptying and postprandial blood pressure does not appear to be linear; in healthy older individuals, the fall in blood pressure increased with escalating rates of intraduodenal glucose infusion between 1–2 kcal/min but did not differ between 2 and 3 kcal/min [17]. This apparent “threshold” may reflect concurrent changes in the secretion of gut-derived peptides involved in the regulation of postprandial cardiovascular function. Interventions that slow gastric emptying, either dietary (e.g., co-ingestion of dietary fibre, guar gum) or pharmacological (e.g., acarbose and GLP-1 receptor agonists), have been shown to attenuate the fall in SBP in response to a carbohydrate meal in “healthy” older individuals and those with T2D [18,19,20].

The interaction of nutrients with the small intestine results in splanchnic vasodilatation to facilitate the absorption of nutrients via the portal circulation. In parallel with this phenomenon, interventions that slow the rate of small intestinal carbohydrate absorption, such as ingestion of an alpha-glucosidase inhibitor, acarbose [21], or small intestinal infusion of a viscous polysaccharide, guar gum [22], are associated with reduced splanchnic blood pooling and attenuation of the fall in SBP in “healthy” older individuals. Variations in nutrient absorption along the small intestine affect the region of the intestine exposed to nutrients, and this may also influence postprandial haemodynamics. In a recent study, a standardised glucose infusion (2 kcal/min over 60 min) was delivered into the duodenum (13 cm post-pylorus) or ileum (190 cm post-pylorus) in individuals with T2D, and changes in blood pressure, heart rate and superior mesenteric artery flow were evaluated. Duodenal glucose exposure was shown to result in a much greater decline in SBP and rise in superior mesenteric artery flow than ileal [23], in association with more rapid glucose absorption, greater GIP release, and less GLP-1 secretion [24]. The latter observation supports the influence of gut-derived peptides in the regulation of postprandial cardiovascular function (as discussed in Section 3.1, Section 4.1 and Section 5.1).

The fall in systemic blood volume secondary to splanchnic vasodilatation is normally compensated for by a combination of enhanced cardiac output via increases in heart rate and/or stroke volume, and increased systemic vascular resistance [25]. Multiple neurohormonal mechanisms have been implicated in the cardiovascular response to a meal. Gastric distension, such as with meal ingestion, triggers a “gastrovascular reflex,” involving the stimulation of noradrenaline secretion, which enhances sympathetic nervous activity. This response is often blunted in the elderly, particularly those with PPH [2]. A number of vasoactive gut peptides have recently been implicated in the regulation of postprandial cardiovascular function, most notably GLP-1, GIP and somatostatin. Early interventional studies suggest that modulation of the secretion or signalling of these gut peptides may have a profound impact on the blood pressure response to meals, providing potential novel targets for the management of PPH (as discussed in Section 3.1, Section 4.1 and Section 5.1) [24,26]. Other gut peptides, including amylin, calcitonin-gene-related peptide, neurotensin, vasoactive-intestinal peptide, bradykinin and substance P are conceivable targets for modulating postprandial cardiovascular function, either via slowing of gastric emptying [27] or vasoactive actions [1]. However, there is a lack of evidence to support their role in postprandial cardiovascular function [1,28].

## 3. Glucagon-Like Peptide-1 (GLP-1)

GLP-1 is secreted by enteroendocrine L-cells which are located most densely in the ileum and colon. The secretion of GLP-1 is minimal during fasting, but is increased markedly following intestinal nutrient stimulation, particularly when the distal gut is exposed [29]. The majority of GLP-1 is inactivated prior to reaching the peripheral circulation by dipeptidyl peptidase-4 (DPP-4), an enzyme located on the surface of endothelial cells in close proximity to enteroendocrine cells [30], as well as in the liver and within the circulation [31]. Despite its rapid degradation, GLP-1 mediates considerable postprandial insulin secretion via the “incretin effect”, i.e., enhanced insulin secretion following oral or enteral glucose loads, when compared with an “isoglycaemic” intravenous glucose infusion [24]. In addition, GLP-1 slows gastric emptying and suppresses glucagon secretion [24]. Accordingly, both the DPP-4 resistant GLP-1 receptor agonists (GLP-1RAs) and DPP-4 inhibitors have been developed for glycaemic control in T2D. It is noteworthy that augmented GLP-1 secretion may also underlie the anti-diabetic effect of older anti-diabetic agents, such as metformin [32,33] and alpha-glucosidase inhibitors [34,35,36], since they delay intestinal glucose absorption and, hence, increase stimulation of the enteroendocrine L-cells in more distal gut regions (Table 1). Since its discovery in 1987 [37,38], numerous extra-glycaemic actions of GLP-1 have been appreciated, including effects on the cardiovascular system. The clinical implications of the latter remain to be explored comprehensively.

### 3.1. Effects of GLP-1 on Postprandial Haemodynamics

**Figure 2:**
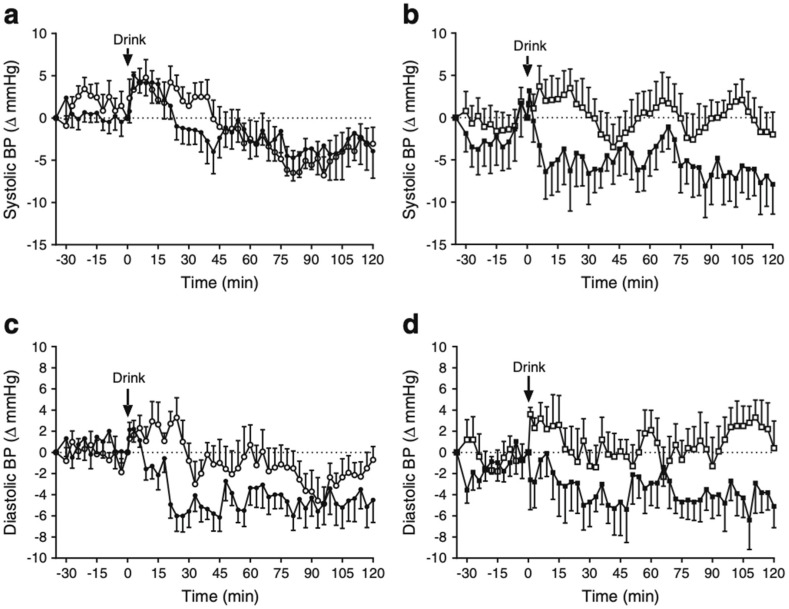
Effect of intravenous GLP-1 (0.9 pmol/kg/min; open symbols) vs saline (filled symbols) on SBP and DBP before and after 75 g oral glucose in “healthy” older (*n* = 14; (**a**,**c**)) and T2D (*n* = 10; (**b**,**d**)) subjects. Data are mean values ± standard error of the mean. Intravenous GLP-1 increased DBP in “healthy” older subjects (*p* < 0.001), and SBP and DBP in T2D subjects (*p* < 0.05 for both), postprandially [7].

In rodents, intravenous administration of GLP-1 was shown to increase sympathetic activation, evidenced by a thermogenic effect attenuated by adrenalectomy or pre-treatment with pharmacological antagonists of sympathetic nervous activity [40]. In humans, GLP-1RAs do not affect preprandial blood pressure acutely [41], but have been shown to reduce SBP modestly in the long-term, an effect attributed to enhanced natriuresis and weight loss [42]. Few studies however, have attempted to discriminate between the cardiovascular profiles of GLP-1 in the fasting and postprandial states [43]. Intravenous GLP-1 administration at a pharmacological dose (0.9 pmol/kg/min) attenuates the fall in SBP and DBP (~5 mmHg at 60 min) in response to a carbohydrate meal in T2D (Figure 2) and does so variably in “healthy” older individuals [3,7]. This occurs in association with slowing of gastric emptying, a reduction in superior mesenteric artery blood flow, and a variable increase in heart rate [3,7]. GLP-1 may have direct effects on cardiac pacemaker cells given that cardiac GLP-1 receptors appear to be localised to the atria, where the sinoatrial and atrioventricular nodes reside [24]. Accordingly, the release of GLP-1 from the distal gut may logically serve as a “negative feedback” mechanism to prevent an exacerbated hypotensive response to increased delivery of nutrients into the small intestine.

Paradoxically, in one study, higher plasma GLP-1 levels were associated with PPH in patients with multiple system atrophy, but it remains uncertain as to whether increased GLP-1 levels were a cause of PPH or secondary to multiple system atrophy. Notably, the individuals with PPH in this study had sympathetic failure which may have attenuated the effect of GLP-1-signalling on blood pressure [44].

### 3.2. Interventional Strategies Utilising GLP-1 for Postprandial Blood Pressure Control

The potential for GLP-1 to modulate postprandial cardiovascular function has rendered it an attractive target for maintaining postprandial blood pressure. Several antidiabetic agents, including GLP-1RAs, DPP-4 inhibitors, metformin and alpha-glucosidase inhibitors, have been investigated for their effects on postprandial blood pressure and GLP-1.

**Figure 3:**
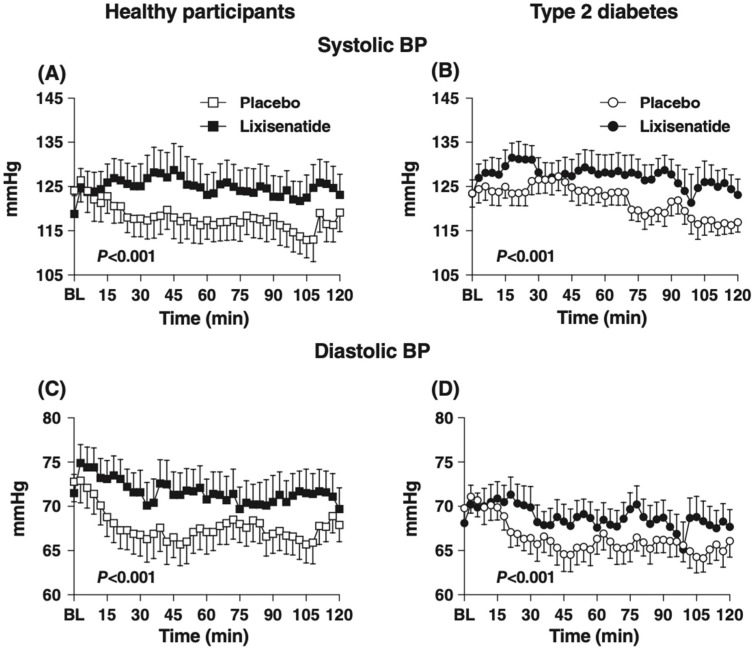
Effects of lixisenatide (10 μg subcutaneously), compared with placebo, on SBP (**A**,**B**) and DBP (**C**,**D**) immediately before and after a 75 g glucose drink, in individuals with (**B**,**D**) and without T2D (**A**,**C**). Data are mean values ± standard error of the mean. Lixisenatide attenuated the fall in SBP and DBP in both groups [20].

The short-acting GLP-1RAs, such as exenatide BD and lixisenatide, have demonstrated similar effects to administration of exogenous GLP-1 on the postprandial haemodynamic profile. In individuals with and without T2D, administration of lixisenatide (10ug, by subcutaneous injection) markedly slowed gastric emptying and attenuated the increase in superior mesenteric artery flow and fall in SBP and DBP postprandially (Figure 3) [20]. Acute administration of exenatide BD increased heart rate and attenuated the fall in SBP and DBP in response to intraduodenal glucose infusion (2 kcal/min) in patients with T2D [45]. In contrast, the effects of long-acting GLP-1RAs, such as exenatide QW and semaglutide, on postprandial haemodynamics are less well-studied. Even though sustained stimulation of the GLP-1 receptor is known to be associated with tachyphylaxis for the slowing of gastric emptying by GLP-1 [46], there is recent evidence that exenatide QW [47] and semaglutide [48] may retain some capacity to slow gastric emptying in health and T2D with prolonged use. Hence, even long-acting GLP-1RAs may have the potential to attenuate the postprandial fall in blood pressure.

The cardiovascular effects of DPP-4 inhibitors (“gliptins”) are of increasing interest [49]. There is limited information about the effects of DPP-4 inhibition on postprandial blood pressure. Reported benefits of DPP-4 inhibitors on PPH relate primarily to case studies, as well as a small cohort of overweight T2D patients receiving metformin therapy [50,51,52]. For example, in a comparative study in overweight patients with T2D, 8 weeks treatment with the DPP-4 inhibitor, linagliptin, while achieving comparable glucose-lowering to glimepiride (a sulphonylurea anti-diabetic drug which does not affect haemodynamics), was reported to attenuate the fall in blood pressure after a meal (0.7 ± 2.3 mmHg) without impacting preprandial blood pressure [50]. In contrast, when another DPP-4 inhibitor, vildagliptin, was administered acutely with an intraduodenal glucose infusion in T2D, postprandial SBP and DBP were lower when compared with placebo [53]. In T2D, sitagliptin administration did not significantly impact blood pressure after a potato meal [54]. The discrepancy in the studies of vildagliptin and sitagliptin, compared with linagliptin, may relate to differences in study design, including the study duration and method of carbohydrate administration. Perhaps the most important difference in the studies was the concomitant use in the former of metformin, a drug known to moderate cardiovascular outcomes with DPP-4 inhibitors and potentially act to augment DPP-4 inhibition to enhance plasma active GLP-1 levels [55,56].

**Figure 4:**
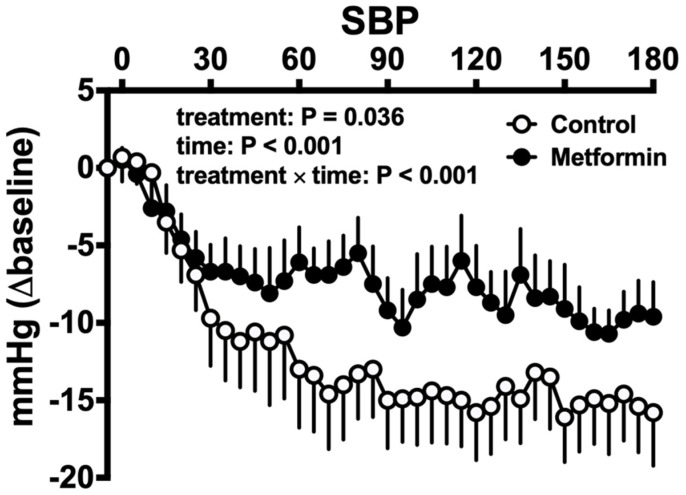
Effect of intraduodenal infusion of metformin 1g, compared with placebo, on the SBP response to 50g oral glucose in T2D. Data are mean values ± standard error of the mean. Metformin attenuated the fall in SBP postprandially [57].

The impact of metformin on postprandial blood pressure has only been evaluated in a small cohort of T2D patients. In this study, intraduodenal infusion of metformin 1g attenuated the fall in blood pressure after 50 g oral glucose substantially (by almost 10 mmHg 60 min after a meal) (Figure 4), an effect associated with an increase in plasma GLP-1 and slowing of gastric emptying [57]. Metformin has also been shown to slow intestinal glucose absorption and increase noradrenaline secretion [58], which may contribute to the attenuation of the fall in blood pressure after enteral glucose.

**Figure 5:**
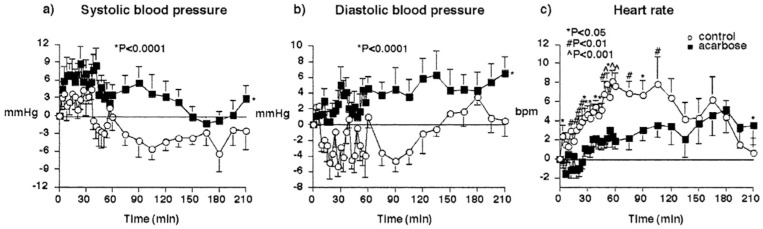
Effect of 100 mg acarbose (black squares) vs. placebo (open circles) on the SBP (**a**), DBP (**b**) and heart rate (**c**) responses to 100 g sucrose in “healthy” older subjects. Data are mean values with 95% confidence intervals. Acarbose increased SBP and DBP, and reduced heart rate postprandially [19].

Observations on the postprandial haemodynamic effects of alpha-glucosidase inhibition have focused primarily on acarbose, with minimal study of the other drugs of this class [59]. Acarbose administration in “healthy” individuals leads to enhanced plasma GLP-1, although this is inconsistent in individuals with T2D [19,36,60]. Several small cohorts [61,62,63] and case studies [64,65,66,67] support the potential for acarbose to modulate postprandial haemodynamics. In one such case study, a 58-year-old individual with T2D, complicated by microvascular disease and severe symptomatic postprandial hypotension, received acarbose (300 mg/day), octreotide and midodrine on separate days, undergoing 24 h ambulatory blood pressure monitoring. Postprandial blood pressure, along with orthostatic dizziness and postprandial vertigo, were attenuated after acarbose, but not octreotide or midodrine [64]. The acute effect of a single oral dose of acarbose (50–100 mg) on blood pressure and heart rate after a standardised meal has been studied in cohorts of “healthy” elderly (Figure 5) [19,63], T2D with PPH [62], and subjects with pure autonomic failure [61]. In each case, the fall in postprandial blood pressure was attenuated significantly (by 15–20 mmHg SBP). Postprandial heart rate was affected by acarbose in a single study (Figure 5). The effect of sustained, relative to acute, acarbose administration on postprandial blood pressure is less well-studied, although case studies suggest it is maintained [67,68]. Slowing of gastric emptying, which may result from enhanced GLP-1 concentrations, and attenuation of postprandial splanchnic vasodilation with acarbose, are likely to contribute to its effects on postprandial haemodynamics [21,69]. Practically, the widespread use of acarbose is not limited by safety or cost, but by gastrointestinal adverse effects, including flatulence and diarrhoea, which are common but tend to subside with continued treatment, and can be minimised through stepwise dose increments and dietary modifications (consuming complex carbohydrate over simple sugars) [70,71].

These therapies, originally introduced as glucose-lowering agents, offer exciting therapeutic potential for the management of PPH, but further studies in larger cohorts of patients with and without T2D are warranted.

## 4. Glucose-Dependent Insulinotropic Polypeptide

GIP is the first of the two “incretin hormones” identified in the 1970s [72]. It is released from the enteroendocrine K-cells which predominate in the proximal small intestine. Like GLP-1, GIP has a short half-life in the systemic circulation due to prompt inactivation by DPP-4. Plasma GIP concentrations are low in the fasting state and increase promptly in response to nutrient stimulation [24]. As an incretin hormone, GIP has a well-established effect to stimulate postprandial insulin secretion in a glucose-dependent manner [73]. Unlike GLP-1, GIP has little effect on gastric emptying, and can augment glucagon secretion in the face of falling glycaemia. It is increasingly recognised that GIP exhibits numerous extraglycaemic functions mediated by GIP receptors identified in diverse tissues [24].

### 4.1. Effects of GIP on Postprandial Haemodynamics

GIP has been reported to induce splanchnic blood pooling and increase heart rate, with variable effects on blood pressure [4,5]. Pre-clinical studies have delineated the variable profile of GIP in the splanchnic, compared with systemic, circulation. Intravenous administration of GIP at supraphysiological doses consistently increased splanchnic blood flow in multiple animal studies [74,75,76]. In contrast, intravenous GIP in dogs reduced hepatic artery blood flow [76]. In humans, the postprandial rise in plasma GIP in individuals with T2D receiving sitagliptin concurrently with enteral glucose infusion was proportional to the increase in heart rate [5]. In patients with type 1 diabetes who underwent hyperglycaemic and hypoglycaemic clamps, intravenous GIP infusion was associated with an elevation in heart rate (10.1 ± 2.6 and 16 ± 4.7 bpm respectively), with a concurrent reduction in DBP (5.4 ± 4.5 and 9.7 ± 6.6 mmHg respectively), although SBP was observed to be increased during the former (5.6 ± 3.1 mmHg), and unaffected in the latter, setting [8]. During a hyperglycaemic clamp in T2D patients, GIP infusion led to a reduction in mean arterial blood pressure (10–15 mmHg) and increase in heart rate (~8 bpm) [4]. Splanchnic blood flow was not assessed in these studies but, conceivably, the increase in heart rate represented a compensation for GIP-induced splanchnic blood pooling. Alternatively, given the presence of GIP receptors in the heart [24], GIP could have a positive chronotropic action either directly, or mediated via anti-cholinergic activity [8]. Overall, GIP has been associated with a decline in postprandial blood pressure in most settings, an effect likely mediated via splanchnic blood pooling, at times compensated for by an increase in heart rate.

### 4.2. Interventional Strategies Utilising GIP for Postprandial Blood Pressure Control

Given that GIP enhances splanchnic blood pooling, the impact of GIP agonists and antagonists on PPH would be of interest. A dual GLP-1/GIP agonist, tirzepatide, has been developed for the treatment of T2D [77,78], while GIP antagonism, utilising a selective competitive inhibitor (GIP fragment GIP(3–30)NH2), has been examined in a pre-clinical setting for potential benefits in T2D [79]. Conceivably, GIP agonism could aggravate, and antagonism could benefit, postprandial blood pressure control. Further studies evaluating the effects of such drugs on the cardiovascular system pre- and postprandially are warranted.

## 5. Somatostatin

Originally named “growth-hormone release-inhibiting hormone” upon its discovery in 1973 [80], the hormone now known as somatostatin comprises two main forms—somatostatin-14 and somatostatin-28. The former is dominant in the CNS, arising from the arcuate and anterior periventricular nuclei of the hypothalamus, as well as pancreatic islet delta-cells. The latter is dominant in the gastrointestinal tract, which is the predominant source of somatostatin in humans, being secreted by D-cells throughout the length of the gastrointestinal mucosa [81,82]. The half-life of somatostatin is only 1–3 min and, as a result, it has been considered to act locally to inhibit secretion of various hormones (e.g., insulin, glucagon, secretin, growth hormone) and gastrointestinal fluids (e.g., gastric acid, bile, colonic fluid) [83]. Synthetic analogues of somatostatin with longer half-lives are utilised in clinical practice, including first-generation (octreotide and lanreotide) and second-generation (pasireotide) agents, for a variety of indications (e.g., acromegaly, neuroendocrine tumours, bleeding oesophageal varices) given the effects of somatostatin to inhibit secretion of other hormones and influence splanchnic blood flow (as discussed in Section 5.1) [82]. The relatively high cost and rates of gastrointestinal adverse effects with somatostatin analogues limit their use, although the latter tend to subside with continued use [84].

### 5.1. Interventional Strategies Utilising Somatostatin for Postprandial Blood Pressure Control

Increasing evidence over the past two decades supports a therapeutic role for somatostatin in postprandial hypotension. Octreotide attenuates the postprandial fall in blood pressure in normotensive and hypertensive elderly (by 7–15 mmHg SBP) [9], and autonomic failure with and without diabetes (by 15 ± 2 mmHg SBP) [85,86]. This effect has been attributed primarily to splanchnic vasoconstriction. However, the mechanism(s) by which somatostatin, or its analogues, induce splanchnic vasoconstriction remain poorly understood. This effect is unlikely to be mediated via the autonomic nervous system given that it occurs independently of changes in plasma catecholamine concentrations, and is maintained in autonomic failure [87]. Splanchnic vasoconstriction was shown to occur concurrently with increasing forearm vascular resistance when octreotide was administered to patients with autonomic failure, such that a direct vasopressor effect of the drug appears likely [6,26], although this finding was not replicated in another study [86]. Alternatively, the underlying mechanism for splanchnic vasoconstriction could be neurohumoral. Somatostatin and its analogues inhibit the secretion of a number of gut peptides, including glucagon [88] and GIP [89], both of which are known to induce splanchnic blood pooling. However, markedly greater glucagon concentrations than those occurring following octreotide are required to induce this effect [88].

While octreotide is one of the most well-studied agents for PPH, few studies have examined the effect of sustained exposure. Ludwig et al. reported a reduction in splanchnic vasoconstriction after 48 h of octreotide use in “healthy subjects,” raising concern of tachyphylaxis [88]. However, attenuation of the fall in postprandial blood pressure was sustained with use of octreotide over a 6 month period in patients with multiple system atrophy [88,90].

## 6. Conclusions

PPH is an important and under-recognised clinical phenomenon associated with increased morbidity and mortality, and intrinsically linked to gastrointestinal function. Gut peptides, most notably GLP-1, GIP and somatostatin, are of particular interest as potential pharmacotherapy targets for PPH. GLP-1 receptors are the target of several existing drugs prescribed for glucose-lowering in T2D which, based on studies of small-cohorts, offer potential as therapies for PPH, extending their application beyond the management of T2D. Clinical trials involving sustained administration of these medications in large cohorts, with or without T2D, to document their effects on postprandial blood pressure, and the complications of PPH, are warranted. GIP may both contribute to the fall in postprandial blood pressure, and the haemodynamic effects of GIP receptor agonists and antagonists postprandially should also be examined. Somatostatin attenuates the fall in postprandial blood pressure, and its analogue, octreotide, has already been studied as a pharmacological therapy for PPH. Discrimination of the cardiovascular effects of gut peptides and their signalling pathways before and after meals may refine the therapeutic approach to PPH.

## Figures and Tables

**Table 1:** Pharmacotherapies with GLP-1 based actions and the mechanisms of their effects [24,33,34,35,36,39].

Drug Class	Example Drugs	GLP-1 Associated Effect(s)
Biguanide	Metformin	↑ secretion (directly/indirectly) ↓ DPP-4 activity (modest)
Alpha-glucosidase inhibitor	Acarbose Miglitol Voglibose	↑ secretion ↓ DPP-4 activity (voglibose)
Short-acting GLP-1RA	Exenatide BD Lixisenatide	Activation of GLP-1 receptors
Long-acting GLP-1RA	Dulaglutide Exenatide QW Liraglutide Semaglutide	Activation of GLP-1 receptors
DPP-4 inhibitor	Alogliptin Linagliptin Sitagliptin Saxagliptin Vildagliptin	↑ intact GLP-1 plasma half-life

↑: increase; ↓: decrease; GLP: glucagon-like peptide; DPP: dipeptidyl peptidase.

## Data Availability

The data presented in this study are openly available in PubMed.
